# Oral Supplementation with a Special Additive of Retinyl Palmitate and Alpha Tocopherol Reduces Growth Retardation in Young Pancreatic Duct Ligated Pigs Used as a Model for Children Suffering from Exocrine Pancreatic Insufficiency

**DOI:** 10.3390/ijms17101642

**Published:** 2016-09-28

**Authors:** Anne Mößeler, Marion Schmicke, Martin Höltershinken, Martin Beyerbach, Josef Kamphues

**Affiliations:** 1 Institute for Animal Nutrition, University of Veterinary Medicine Hannover, Foundation, Bischofsholer Damm 15, D-30173 Hanover, Germany; josef.kamphues@tiho-hannover.de; 2 Clinic for Cattle, University of Veterinary Medicine Hannover, Foundation, Bischofsholer Damm 15, D-30173 Hanover, Germany; marion.schmicke@tiho-hannover.de (M.S.); martin.hoeltershinken@tiho-hannover.de (M.H.); 3 Department for Biometry, Epidemiology and Information Processing, University of Veterinary Medicine Hannover, Foundation, Bünteweg 2, D-30559 Hanover, Germany; martin.beyerbach@tiho-hannover.de

**Keywords:** alpha-tocopherol, animal model, cystic fibrosis, fat-soluble vitamins, growth, leptin, pancreatic exocrine insufficiency, pigs, retinyl palmitate

## Abstract

Pancreatic exocrine insufficiency (PEI) is a disease of diverse aetiology—e.g., majority of patients suffering from cystic fibrosis (CF) show PEI congenitally. Malnutrition and malabsorption of nutrients impair growth and nutritional status. As reduced fat digestion leads to a deficiency of fat-soluble vitamins the supplementation is standard, but absorption is a critical point in PEI-patients. The pancreatic duct ligated (PL) pig is an established model for PEI in humans and has been proven to be a suitable model to compare different vitamin additives for supplementation. In a former study, PEI caused distinct growth retardation in young piglets, but did not affect growth in older ones. Our study hypothesised that this age-dependent effect is caused by exhausted body reserves of fat-soluble vitamins and, therefore, extra supply reduces growth retardation. PEI was induced by PL at the age of seven (PL-7) or 16 weeks (PL-16). Controls (C) underwent a sham surgery. Some PL-7 pigs (PL-7 + Vit) were fed a special vitamin additive. PEI reduced the mean final body weight (kg) at 26 weeks of age significantly with lower effect in PL-16-pigs (C:117; PL-7:49.5; PL-7 + Vit:77.1; PL-16:96.4). Extra vitamin supply resulted in an increased growth and normalised serum concentration of alpha-tocopherol, underlining the importance of special supplementation in PEI-patients.

## 1. Introduction

Pancreatic exocrine insufficiency (PEI) is a disease occurring in humans, as well as veterinary patients, resulting in maldigestion and malabsorption of nutrients, especially of fat [1]. As a consequence, patients suffering from PEI are at high risk of developing a deficiency of fat-soluble vitamins [2,3,4,5]. Fat-soluble vitamins are essential as they have many functions (antioxidant, coenzyme, neurodevelopment, bone development, coagulation [6,7]). Therefore, deficiency has manifold effects. As most patients (~90%) with cystic fibrosis (CF) suffer from PEI [8,9], mostly congenitally, fat-soluble vitamin status is of special interest in these patients. A fat-soluble vitamin deficiency can occur even if patients are treated with pancreatic enzyme replacement therapy [10]. Deficiencies of vitamin A, D, and E are known to be present in PEI-patients for a long time [11,12]; and were detected even in very young (<2 months) children [13]. Blood values decrease with age in CF patients, with adult patients showing the highest prevalence of vitamin E deficiency [14]. Due to routine pancreatic enzyme replacement therapy and vitamin supplementation clinical manifestation of vitamin deficiency is uncommon nowadays [15]. Nevertheless, low blood levels of fat-soluble vitamins (particularly A, E, and K) are common in CF patients [16]. Consequently, today there is a general recommendation to routinely supplement fat-soluble vitamins [17,18,19] and to check blood status on a regular basis (at least annually) [20].

The pancreatic duct ligated (PL) pig is an established model for PEI in humans [21,22,23]. As exocrine pancreatic function is essential for digestion and absorption of nutrients the complete loss of pancreatic secretion causes maldigestion and malabsorption of various macro- and micronutrients [24,25,26,27]. The juvenile PL pig was used in several studies to test the effects of PEI on growth and different parameters in juveniles [23,28,29,30,31]. Due to the tremendous growth rate of juvenile pigs (birth weight: 1.5 kg; weight at six months: 120 kg) deficiencies develop much faster than in humans. The literature concerning effects of PEI on growth of young pigs is controversial. While some authors found massive growth retardation [21,23,28,32,33] others found only partial growth retardation [31,34,35]. It was assumed that the age of pigs at experimental induction of PEI is highly relevant for the effects on growth [28]. These authors found complete growth retardation if PEI was induced in young pigs (aged seven weeks), but stated that exocrine pancreatic function is dispensable for growth in pigs aged 16 weeks. As deficiency of fat-soluble vitamins is common in PEI patients and was observed to occur very quickly in juvenile PL pigs, even the vitamin concentration of the diet being far (six- to eight-fold) above recommended values for healthy pigs [36,37], it was speculated that the age at induction of PEI might have influenced growth [28] due to a different extent of reserves of fat-soluble vitamins and/or the exhaustion being dependent on duration of PEI. The objective of the present study was to investigate whether an extra supply of fat-soluble vitamins in a water-soluble form, which was proven to be absorbable in juvenile PL pigs treated with pancreatic enzymes [36], reduces growth retardation in these pigs used as a model for children. Repeated measurements of serum concentrations of vitamin A and E were taken during the course of time, as well as concentrations of vitamin A and E in the liver at the end of the trial. This experimental study aimed to prove the relevance of fat-soluble vitamins for growth in juvenile individuals suffering from PEI.

## 2. Results

### 2.1. Body Weight and Body Measurements, Feed Intake, and Body Weight Gain:Feed Ratio

**Figure 1:**
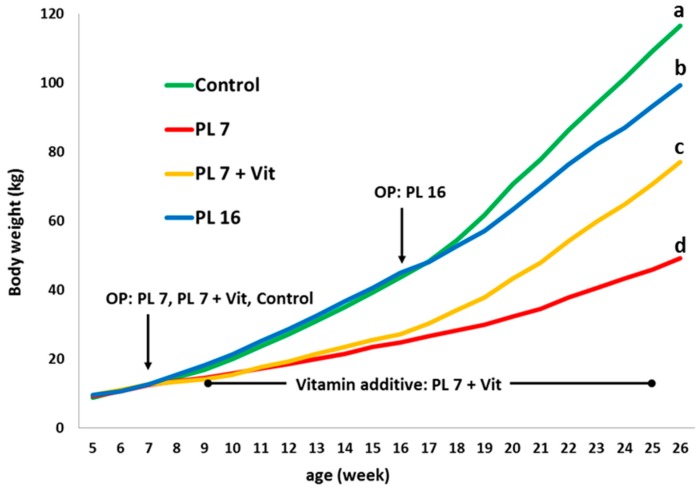
Body weight development of controls and pancreatic duct ligated (PL) pigs during the course of the trial; pancreatic duct ligation was performed in week 7 for PL-7 and PL-7 + Vit (PL 7 pigs receiving oral vitamin additive) or in week 16 (PL-16); OP: surgery; different letters mark significant (*p* < 0.05) differences for group-wise comparison at the end of the trial.

The experimentally-induced PEI reduced growth (final body weight at 26 weeks of age) in both PL-16 and PL-7, with PL-7 being the smallest (*p* < 0.05). Vitamin additive (PL-7 + Vit) resulted in a significantly increased (*p* < 0.05) growth compared to PL-7, but caused no complete normalisation (values neither reached those of PL-16 nor controls; see Figure 1; Table 1). It is worth mentioning that the extra oral vitamin supply did not affect the body weight development directly after introduction of this supplement in PL-7 + Vit in week nine of life. Distinct effects of the vitamin additive on growth were found from week 17 onwards. For the group PL-16 a reduced growth rate was detected from week 19 of life onwards (see Figure 1).

For body length (nose to tail length), again, PL-7 showed the lowest values, while the length of PL-16 differed neither from the controls nor PL-7 + Vit (*p* > 0.05). In the latter group the body length was significantly higher (*p* < 0.05) compared to PL-7. The metacarpus perimeter did not differ significantly between PL-16 and controls, whereas the lowest values were found in PL-7 (*p* < 0.05). The group of PL-7 pigs supplemented with vitamins (PL-7 + Vit) showed significantly higher values (*p* < 0.05) than PL-7 but did not reach the values of controls or PL-16. Absolute feed intake during the ad libitum phase was lowest in PL-7 but when body weight was taken into account daily feed intake was lower (*p* < 0.05) in controls.

Body weight (BW) gain:feed ratio (G:F) during the entire study was highest in controls and lowest in PL-7 (*p* < 0.05). PL-7 + Vit showed higher G:F compared to PL-7 (*p* < 0.05). Nonetheless, the highest G:F in PL pigs was seen in PL-16 pigs (see Table 1). For all body measurements the following sequence emerged: Controls > PL-16 > PL-7 + Vit > PL-7. The relative weight of the gastrointestinal tract (GIT) was higher in PL pigs (*p* < 0.05) with the highest values in PL-7. While in the controls the GIT accounted for only 8% of BW, in PL-7 pigs more than 17% of BW were allotted to GIT. The relative proportion of digesta on BW was significantly increased (*p* < 0.05) in all groups of PL-pigs, reaching values twice as high compared to the controls (see Table 1).

### 2.2. Faecal Fat Digestibility

Fat digestibility at a faecal level was massively reduced (*p* < 0.05) by pancreatic duct ligation but did not differ (*p* > 0.05) between different groups of PL pigs (Control: 79.0 ± 2.00; PL-7: 14.3 ± 9.20; PL-7 + Vit: 14.3 ± 14.4; PL-16: 13.0 ± 10.9).

### 2.3. Leptin Concentration in Serum

Leptin concentration in serum (ng/mL) was reduced (*p* < 0.05) in week 26 of life in all groups of PL pigs compared to controls (Control: 14.0 ± 8.69; PL-7: 0.673 ± 0.798; PL-7 + Vit: 0.919 ± 1.04; PL-16: 1.16 ± 1.59). There was neither any effect of age at induction of PEI nor extra vitamin supply as levels of serum leptin did not differ significantly between groups of PL-pigs.

### 2.4. Serum Levels of Vitamin A (Retinol) and Vitamin E (Tocopherol)

**Figure 2:**
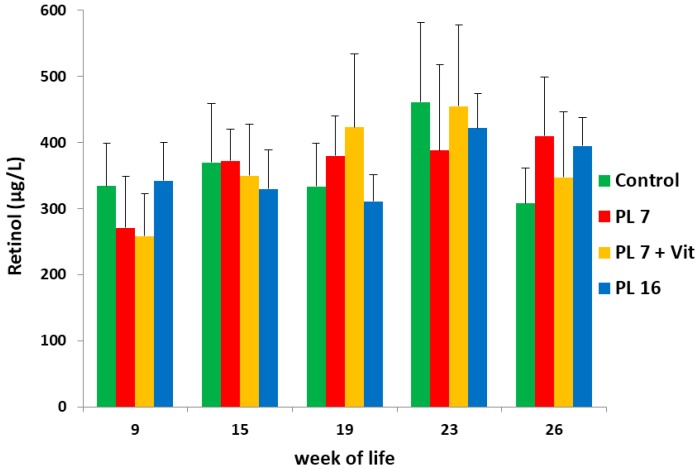
Vitamin A (retinol) concentration (µg/L) in serum of controls and PL pigs during the course of the trial; pancreatic duct ligation was performed in week 7 for PL-7 and PL-7 + Vit or in week 16 (PL-16).

Mean serum concentrations of vitamin A did not differ between the groups (*p* > 0.05), varying within the reference range [38] throughout the entire course of the study (see Figure 2).

**Figure 3:**
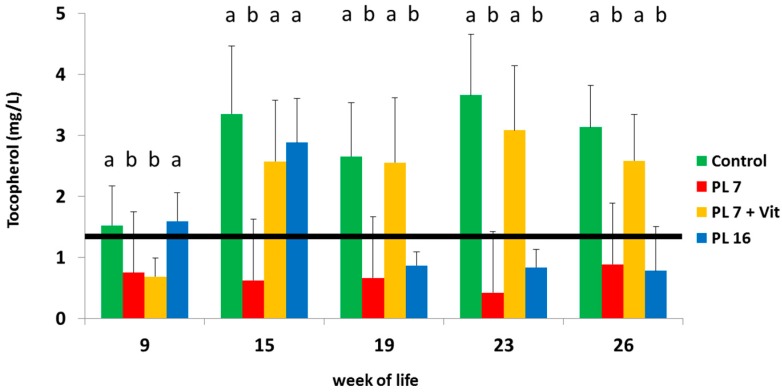
Vitamin E (tocopherol) concentration (mg/L) in serum of controls and PL pigs during the course of the trial; pancreatic duct ligation was performed in week 7 for PL-7 and PL-7 + Vit or in week 16 (PL-16); different superscripts mark significant (*p* < 0.05) differences for group-wise comparison at the same time; black horizontal line marks lower reference value.

Serum concentrations of vitamin E were above the lower reference range (1.2 mg/L [38]) during the entire study in the control animals. In all pigs with pancreatic duct ligation at week 7 of life the vitamin E concentration was below the lower reference range at nine weeks of age (see Figure 3). Furthermore, serum vitamin E concentrations were markedly reduced and below the reference range from week 19 onwards in group PL-16. Adding the vitamin additive (beginning in week 9) resulted in a marked rise (*p* < 0.05) of vitamin E concentration in serum with levels being within the reference range and not differing (*p* > 0.05) from those of the controls (see Figure 3).

### 2.5. Concentrations of Vitamin A (Retinol) and Vitamin E (Tocopherol) in Liver Tissue at the End of the Trial

**Figure 4:**
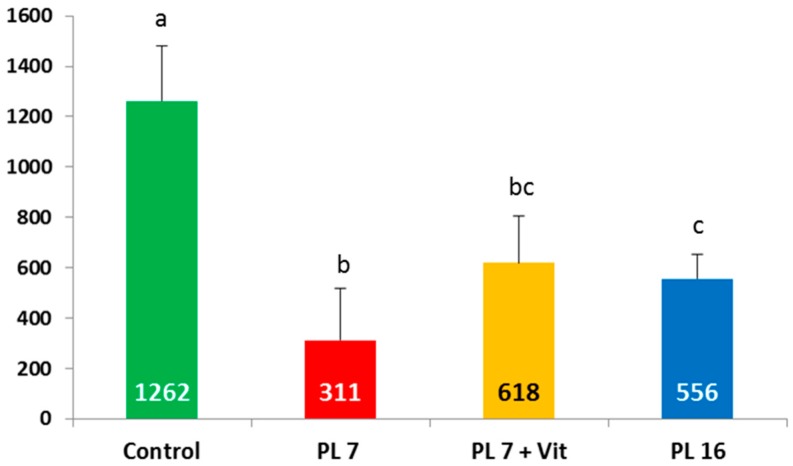
Vitamin A (retinol) concentration (mg/kg dry matter) in liver tissue of controls and PL pigs at the end of the trial; pancreatic duct ligation was performed in week 7 for PL-7 and PL-7 + Vit or in week 16 (PL-16); different superscripts mark significant (*p* < 0.05) differences for group-wise comparison.

Retinol concentration in liver tissue differed markedly between the different groups (see Figure 4). In all groups with experimentally-induced PEI significantly decreased retinol concentrations were found in the liver tissue. There was a significant effect of age at induction of PEI with pigs of group PL-16 showing a significantly (*p* < 0.05) higher value than pigs in group PL-7. In pigs receiving the vitamin additive orally (PL-7 + Vit), the values were nearly doubled compared to PL-7; nonetheless this difference reached no significance (*p* > 0.05).

**Figure 5:**
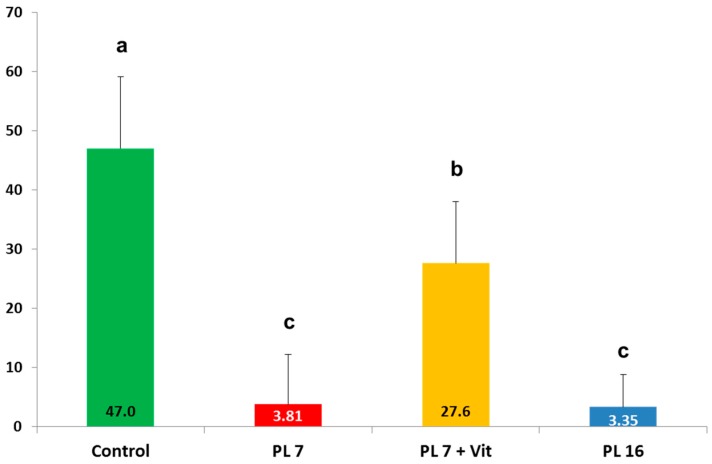
Vitamin E (tocopherol) concentration (mg/kg dry matter) in liver tissue of controls and PL pigs at the end of the trial; pancreatic duct ligation was performed in week 7 for PL-7 and PL-7 + Vit or in week 16 (PL-16); different superscripts mark significant (*p* < 0.05) differences for group-wise comparison.

Tocopherol concentration in liver tissue differed markedly between the different groups (see Figure 5). While experimentally-induced PEI resulted in a distinct drop in values in liver tissue in PL-7 and PL-16, the values observed in the liver tissue of PL-pigs receiving the oral vitamin additive were significantly (*p* < 0.05) higher and within the reference range (15–50 mg/kg dry matter liver tissue; [38]).

## 3. Discussion

Experimentally-induced PEI resulted in a distinct reduction in growth. However, there was no complete growth retardation in PL-7 pigs as had been previously observed [28], or even weight loss, as had been described in earlier studies [23,24,34]. Furthermore, the growth of PL-16 pigs was markedly reduced as well. It seems noteworthy to emphasise that surgery was performed on the animals at the same age in this study as had been the case in the study of Fedkiv et al. [28]. Additionally, the BW of the animals was comparable in both studies (for PL-7 as well as PL-16). Interestingly, the relative weight of the GIT was significantly higher in all groups of PL pigs compared to the controls, thus confirming results of a former study [31]. This higher mass of the GIT results in an overestimation of growth and nutritional status when BW is used as the sole parameter for growth. Taking into account that the vitamin E level of the diet used in this study was eight times higher than recommendations for healthy pigs, the very fast drop in serum levels of the PL pigs is surprising and underlines the clinical relevance of vitamin E supplementation for PEI patients. The fact that the oral supplementation of fat-soluble vitamins resulted in a distinctly higher growth rate in group PL-7 + Vit provides strong evidence that the exhaustion of fat-soluble vitamins [37], especially of vitamin E, is a critical point in juvenile PEI patients and a highly relevant aspect for the growth retardation observed in young PL pigs. Fat digestibility did not differ between groups of PL pigs with or without vitamin additive, indicating that the added emulsifiers did not relevantly affect fat digestibility and the outcome of the study, besides increasing the availability of the added vitamins. The finding concerning serum vitamin A varying within the reference range even in PL-7 pigs during the entire study duration is presumably a result of the mobilisation of resources stored in the animals’ body (e.g., liver; [37]). The lower vitamin A content in the liver of the PL pigs indicates that these animals had released vitamin A from the liver into the circulation to maintain the normal serum concentrations observed during the entire study. In contrast, vitamin E levels in serum declined very fast after experimental induction of PEI, presumably because vitamin E is not stored in the animal‘s body in large amounts [39]. The fact that Fedkiv et al. [28] did not find any effect of PEI on growth in PL pigs that had undergone surgery at week 16 of life might be due to the relatively short duration of that study (and vitamin resources were not exhausted within this time as the animals had been able to build up reserves in earlier life). Furthermore, the maldigestion and malabsorption of nutrients caused by PEI might have masked the reduced growth in that study [28] due to higher gut fill [31,35].

The leptin concentration was reduced in all groups of PL pigs compared to the controls (*p* < 0.05) without any effect of age at induction of PEI or extra vitamin supplementation, indicating that, despite marked (*p* < 0.05) differences in body weight, the body fat content was reduced in all PL pigs. Studies in mice revealed a significant influence of leptin on growth during malnutrition. In these studies it was shown that mice treated with leptin had a greater tibial length than untreated mice and a similar tibial length to control mice fed ad libitum despite the lower weight [40]. Therefore, the low leptin levels observed in PL-pigs may be biologically linked not only to the lower body fat content but also to the lower growth rate. Leptin concentration in serum might be a promising parameter to estimate the nutritional status in patients suffering from PEI indirectly but validation is necessary. The greatest advantage of using a blood parameter like leptin to estimate body fat content is the fact that no expensive and complex equipment is necessary on-site. Therefore, this parameter could be included in routine checks. A more precise characterisation of growth, using not only body weight but also further parameters like body length and metacarpus perimeter (to quantify skeletal growth) and leptin levels in blood, allows a deeper understanding of the processes occurring in the case of PEI.

This study clearly demonstrates the great relevance of sufficient vitamin E supply in the case of PEI for individuals at different ages. Furthermore, the results indicate that pancreatic exocrine function is not dispensable in pigs aged 16 weeks as previously stated [28]. The different outcome regarding the effects of PEI on growth observed in former studies might be a result of varying supplies and/or availability of fat-soluble vitamins. This is demonstrated in the present study where the exhaustion of fat-soluble vitamins, especially vitamin E, can occur very rapidly in fast-growing species, like the pig, in the case of maldigestion of fat despite the implemented basal diet containing vitamin concentrations far above recommended values. The present study indicates that the effects of PEI on vitamin status—especially vitamin E—are of greatest relevance in young growing individuals. The fact that growth retardation was much lower in PL pigs receiving an extra vitamin additive of high bioavailability although fat digestion was not improved clarifies the importance of these micronutrients for growth. The frequent blood sampling enables the detection of the very fast drop in tocopherol. Nonetheless, an even more frequent sampling would have allowed a more precise characterisation of the course of the depletion and presumably would have helped to explain the delay in improvement of growth in pigs of group PL-7 + Vit. Taking into account the reserve capacity of the liver for fat-soluble vitamins [41,42] the question arises whether undertaking vitamin analysis in blood samples once a year (as recommended in most guidelines for human patients; e.g., [20]) is sufficient to detect deficiency promptly in PEI-patients. Additionally, the present study illustrates that the higher gut fill must be taken into account in any case of maldigestion or malabsorption whenever body weight is used as a main criterion for growth. The different parameters used to quantify growth in this study allow deeper and more differentiated insights into effects of PEI. The massive increase of digesta due to maldigestion and malabsorption of nutrients would have led to a massive misinterpretation of growth.

All in all, this animal study underlines the importance of increased vitamin E supply in juvenile patients with PEI to minimise negative effects of deficiency on growth and nutritional status. Taking into account that the basal diet with vitamin content far above recommendations (eight-fold higher vitamin E content than recommendations for healthy pigs) was unable to maintain the serum tocopherol within the reference range, using vitamin additives in an adequate (elevated) dosage and of high bioavailability is crucial for optimizing growth of juvenile PEI-patients. The need to have a differentiated look at the status of the different fat-soluble vitamins observed in human CF patients [43] was seen in this animal model as well. Blood levels can vary within the reference range for one fat-soluble vitamin, while others are below the lower limit. The possibility to investigate liver samples in the animal model allows gaining more precise information about vitamin status [37] since blood concentrations stay relatively constant until liver stores are largely exhausted or filled up [43]. Serum retinol concentrations are, therefore, non-sensitive indicators in the case of subtoxicity or toxicity [44]. If only data for serum concentrations of retinol had been available in this study (as is standard in human patients), no differences between the different treatment groups would have become apparent as serum levels did not differ between the different groups. Therefore, these results underline the limited informative value of serum levels in this respect to detect conditions of under- or oversupply by analysis of blood samples [37]. This finding is of special interest as there are highly concentrated preparations of fat-soluble vitamins available on the market nowadays. It must be borne in mind that these modern products, providing the fat-soluble vitamins in a water-soluble form, guarantee not only efficient supplementation but also present the risk of oversupply and toxicosis [43,44,45,46,47].

Furthermore, this study clearly shows the need to use suitable models to test the availability of vitamin supplements. The animal model of the PL pig is a very valuable model for studying efficacy and availability of orally-administered vitamin products for patients with exocrine pancreatic insufficiency. This is of special interest as the potency of antioxidant effect differs between natural or synthetic sources of vitamin E [48]. This animal model might also help to differentiate the biological effects of various derivatives in PEI patients as specific information in these patients is missing.

To summarise, the results of this study clearly show the crucial role of vitamin E on growth in juvenile individuals suffering from PEI. The very fast drop in serum levels of vitamin E despite high levels in basal diet must be taken into account for dietetic recommendations as well as for experimental studies to ensure that growth is not impaired by deficiency of fat-soluble vitamins, especially vitamin E.

## 4. Material and Methods

All efforts were made to minimise both the stress for the individual animal as well as the numbers of animals used. The procedures used in this study were conducted in accordance with the German Animal Welfare Act and with the European Council Directive of 24 November 1986 (86/609/EEC) and were approved by the Ethics Committee on Animal Welfare of the Hannover District Government, Germany.

### 4.1. Animals

A total of 33 female cross-bred piglets of the same genetic background and age were used in two consecutive trials. The piglets were taken from the farm to the research unit at the age of five weeks. In the first week the animals adapted to the conditions at the Institute for Animal Nutrition of the University of Veterinary Medicine, Hannover, Foundation, Germany. Piglets were housed individually, and trials started when pigs reached six weeks of age and had a mean body weight (BW) of 10.8 ± 1.04 kg.

PEI was successfully induced experimentally by ligation of the ductus pancreaticus accessorius in 16 randomly-selected piglets at the age of seven weeks (PL-7 pigs), as described by Tabeling [49], while the remaining nine pigs were sham operated at the same age and served as controls (C). The animals that underwent surgery had a mean BW of 12.7 ± 1.24 kg in week 7. In eight pigs the pancreatic duct was ligated at the age of 16 weeks (PL-16); at that time these pigs had a mean BW of 45.0 ± 1.56 kg. The PEI was confirmed by measuring the chymotrypsin activity in the faeces 10 days post-surgery (test kit purchased from Immundiagnostik AG, Bensheim, Germany, catalogue No. K6990)—only pigs with a chymotrypsin activity <0.90 U/g faeces were defined and used as PL pigs. In this study, in all pigs undergoing pancreatic duct ligation, the experimental induction of PEI was confirmed by chymotrypsin activity levels in faeces below the critical value mentioned above—with one exception: one pig that underwent pancreatic duct ligation but showed a physiological chymotrypsin concentration was assigned to the control group. No pancreatic enzyme replacement therapy was given to any of the PL pigs during the entire study.

### 4.2. Housing

Animals were housed individually without litter in pens of 3 m^2^. Visual contact and limited social contact were provided by means of railings between stables. Animals had free access to water via nozzle drinkers.

### 4.3. Diet and Feeding

The diet used was a complete diet designed to meet the requirements of growing pigs, but with a much higher supply of fat-soluble vitamins than recommended by GfE [50]. The diet (see Table 2) was based on wheat, barley, soy bean meal, and skimmed milk powder, and was enriched with linseed oil and soybean oil.

The diet was fed over the entire study duration in meal form and dry. During the final 10 days chromium oxide was added to the diet at a dosage of 3.26 g per kg dry matter (DM) to calculate nutrient digestibility by using the marker method. Animals were fed three times a day (pair feeding, amounts progressively increasing); during the final two weeks of the trial the animals were fed ad libitum.

### 4.4. Vitamin Supplementation

From week 9 onwards some PL-7 pigs (*n* = 7; PL-7 + Vit) were supplemented with a special formulation of an additive which was proven to be highly absorbable in PL pigs treated with pancreatic enzyme therapy in parallel [36]. Focus was placed on supplementation of vitamin A and E. The vitamin additive accounted for 90,000 IU vitamin A (retinyl palmitate), 500 IU vitamin D_3_ and 600 mg vitamin E (all rac-alpha tocopherol) per kg DM of the diet and was prepared with highly efficient emulsifiers (E 484; glyceryl polyethylene glycolricinoleate; E 1520 propylene glycol). The vitamin additive was supplied by Miavit (Essen (Oldenburg), Germany) and was mixed each day into the daily ration of the individual pigs.

### 4.5. Parameters and Blood Sampling during the Study Course

Feed intake was recorded daily on an individual basis. Animals were weighed once a week in the morning before feed was offered. Blood samples (serum) were taken regularly during the course of study to investigate the concentration of vitamin A (retinol) and vitamin E (alpha-tocopherol). As the surplus of vitamin D based on the oral additive was rather low, the vitamin D levels were not in the focus of this study. The blood samples were taken from the jugular vein, centrifuged, and serum samples were stored at −20 °C until analysis.

### 4.6. Euthanasia and Dissection

All animals were euthanized at 26 weeks of age. At the day of dissection the body length (from nose to tail basis) and metacarpus perimeter were measured using a measuring tape. Body weight was measured directly before dissection (after feed allowance for at least 4 h). During dissection the gastrointestinal tract (GIT) was removed (liver remained in the carcass) and body weight without GIT was defined as empty body weight. To check whether vitamin supplementation had affected fat digestibility (due to the added emulsifier) fat digestibility was calculated at the faecal level (using digesta taken from the rectum).

### 4.7. Analysis

In the serum samples leptin was measured by means of a porcine specific Leptin ELISA (Sea084Po, cloud-clone corp., Houston, TX, USA). Vitamin analysis was performed using HPLC as described in a former study [36]. The digesta from the rectum was analysed using established methods [51] and digestibility of fat was calculated using the marker method. Due to organisational reasons the vitamin A content of liver tissue was only measured in animals in the second part of this study, while vitamin E content was determined in liver samples of all animals.

### 4.8. Statistical Analysis

The *F*-Test of a one-way ANOVA was used to test the global hypothesis of no differences between the four groups. The Fisher`s Least Significance Difference (LSD)-test with a comparison-wise error rate of *p* < 0.05 was applied to test the pairwise differences. Statistical analyses were performed with SAS^®^ software, Version 9.3 (SAS Inst. Inc., Cary, NC, USA). Differences were stated to be significant in case of *p* < 0.05.

Significant differences (*p* < 0.05) were marked by use of different superscripts. To mark the differences between group results, significant differences were marked with different letters. Groups with identical letters do not differ significantly.

## 5. Conclusions

The impaired growth of juveniles suffering from exocrine pancreatic insufficiency could be prevented partly by application of a high-dose vitamin additive being highly absorbable. Vitamin E seems to be of the greatest relevance in this context.

## Figures and Tables

**Table 1:** Body weight (BW), body length, metacarpus perimeter, feed intake, performance data (body weight gain: feed), and further quantification of proportion of total gastrointestinal tract (GIT), digesta (absolute (kg), and relative to body weight (%)); mean ± SD.

Parameter	Control	PL-7	PL-7 + Vit	PL-16
Body weight (BW), kg	117 ± 8.07 ^a^	46.2 ± 22.4 ^d^	73.5 ± 11.9 ^c^	96.4 ± 9.89 ^b^
Body length, nose to tail, cm	143 ± 5.59 ^a^	115 ± 13.4 ^c^	131 ± 9.58 ^b^	138 ± 6.41 ^a,b^
Metacarpus perimeter, cm	17.0 ± 0.66 ^a^	12.6 ± 1.40 ^c^	14.4 ± 0.79 ^b^	16.2 ± 0.80 ^a^
Feed intake, g as fed/day *	3001 ± 489 ^a^	1483 ± 993 ^b^	2721 ± 824 ^a^	3093 ± 687 ^a^
Feed intake, g as fed/kg BW *	25.5 ± 3.91 ^a^	29.5 ± 10.6 ^a,b^	37.1 ± 9.36 ^b^	33.1 ± 5.29 ^a,b^
BW Gain: Feed ^#^	0.498 ± 0.026 ^a^	0.272 ± 0.062 ^d^	0.337 ± 0.037 ^c^	0.389 ± 0.029 ^b^
Empty body weight, kg	108 ± 7.11 ^a^	40.9 ± 19.0 ^d^	64.7 ± 10.8 ^c^	82.5 ± 8.61 ^b^
Relative mass of GIT, % of BW	7.95 ± 0.86 ^a^	17.3 ± 2.00 ^c^	16.3 ± 2.36 ^b,c^	14.4 ± 2.75 ^b^
Digesta, kg	5.30 ± 1.11 ^a^	4.64 ± 1.98 ^a^	7.41 ± 1.34 ^b^	8.42 ± 1.61 ^b^
Digesta, % of BW	4.49 ± 0.78 ^a^	9.53 ± 1.35 ^b^	9.76 ± 1.88 ^b^	8.72 ± 1.34 ^b^

* Week 25 of life during ad libitum feeding phase; ^#^ body weight gain (kg) per kg feed intake; calculated over the entire study period; different superscripts mark significant (*p* < 0.05) effect of group.

**Table 2:** Chemical composition of the experimental diet.

Chemical Composition (per kg Dry Matter)			
Crude ash (g)	55.4	Cu (mg)	17.8
Crude protein (g)	198	Zn (mg)	148
Crude fat (g)	111	Se (mg)	0.449
Starch (g)	400	Vitamin A (IU)	13,362
Ca (g)	11.6	Vitamin D (IU)	1850
P (g)	6.59	Vitamin E (mg)	123

## Abbreviations

BW	Body weight
C	Control group
CF	Cystic Fibrosis
DM	Dry matter
G:F	Gain:Feed ratio
*p*	*p*-Value
PEI	Pancreatic exocrine insufficiency
PL	Pancreatic duct ligated
PL-7	Pigs that underwent pancreatic duct ligation in week 7 of life
PL-7 + Vit	Pigs that underwent pancreatic duct ligation in week 7 of life and received oral vitamin supplement from week 9 of life onwards
PL-16	Pigs that underwent pancreatic duct ligation in week 16 of life
